# Supplementary material for: Impact of Liver Inflammation on Bile Acid Side Chain Shortening and Amidation
Source: Cells. 2022 Dec 9;11(24):3983. doi: 10.3390/cells11243983 (PMC9777420; doi:10.3390/cells11243983)
Supplement: Supplementary file 1 [file cells-11-03983-s001.zip › Supplementary Tables.pdf]

## SUPPLEMENTARY TABLES

**Supplementary Table S1.** Primer sequences used to quantify human gene expression by RT-qPCR.

| Gene | Protein | Accession number | Sequence (5′-3′) | Type |
|---|---|---|---|---|
| *ABCB11* | BSEP | NM_003742 | GTGAAGGAATGGTGACCGTGGA | F |
| | | | TCCTTGGCAGCTTGGACTATGTCT | R |
| *ABCD3* | PMP70 | NM_002858.4 | CGGCCTGCACGGTAAGAAAAGT | F |
| | | | CTTGTCCACCACAGCTCGCT | R |
| *ACOX1* | ACOX1 | NM_004035.7 | CCTGGTGGGCTTGGAAAGACTT | F |
| | | | GTCACCAACGGTAATTCCTGGCA | R |
| *ACOX2* | ACOX2 | NM_003500.4 | CACCCCACTGCCAGGAATCAT | F |
| | | | GCAAGACCTGTGCAAAGCGACT | R |
| *ACOX3* | ACOX3 | NM_003501.3 | CCAAGTACCTCCTCCATAGCTTGGT | F |
| | | | CGTGGCTTAATTCGGTCAGAGCAAA | R |
| *AMACR* | AMACR | NM_014324.6 | GTGGTGGCCTTATGTGTGCACT | F |
| | | | GCTGTTCCTTCCACCATATTTGCATCA | R |
| *ANGPTL4* | ANGPTL4 | NM_139314.3 | AGCAGGATCCAGCAACTCTTCCA | F |
| | | | CCAGGAGGCCAAACTGGCTTT | R |
| *BAAT* | BAAT | NM_001701 | GGGCAGGTTCTCTTGCTAGGTTTT | F |
| | | | CTGGCTCATCAACAAGTGCACTCA | R |
| *CYP7A1* | CYP7A1 | NM_000780.2 | CCATAAGGTGTTGTGCCACGGAAA | F |
| | | | GCCCAAATGCCTTCGCAGAAG | R |
| *FGF19* | FGF19 | NM_005117.3 | GCGCACAGTTTGCTGGAGATCA | F |
| | | | AGTCTTCCTCCGAGTACTGAAGCAG | R |
| *HPRT1* | HPRT1 | NM_000194 | ATGGACAGGACTGAACGTCTTGCT | F |
| | | | TGAGCACACAGAGGGCTACAATGT | R |
| *HSD17B4* | DBP | NM_001292027.1 | TGTTTCAATGAATGATTTGGGAGGGGACT | F |
| | | | TCTTCCACTGAATCATAGTTGGCCACT | R |
| *NR0B2* | SHP | NM_021969 | GGCTTCAATGCTGTCTGGAGT | F |
| | | | CTGGCACATCGGGGTTGAAGA | R |
| *SLC51A* | OST$\alpha$ | NM_152672 | TTCCAGGTTCTCCTCATCCTGAC | F |
| | | | CAATTCATCACTTGAGACCTGGTTTT | R |
| *SCP2* | SCPx | NM_002979.5 | GGGTTGGCATGACCAAGTTTGTGA | F |
| | | | GCTAAAGCCTTCTTGCCTGCTTCTT | R |
| *SREBPF1* | SREBP-1c | NM_001321096.2 | GCACTTTCGAAGACATGCTTCAGCTT | F |
| | | | CTCCCAGCATAGGGTGGGTCAAATA | R |

Forward (F) and reverse (R) primers used in qPCR.

**Supplementary Table S2.** Primary antibodies and conditions for immunoblotting.

| Antigen | Origin | Dilution | Blocking proteins | Reference |
|---|---|---|---|---|
| BAAT | *Oryctolagus cuniculus* | 1:1000 | Milk 5% | Novus Biologicals (NBP2-92503) |
| OSM | *Capra aegagrus hircus* | 1:500 | BSA 5% | R&D systems (BAF295). |
| pY705-STAT3 | *Mus musculus* | 1:1000 | BSA 5% | Cell Signaling Technology (9138) |
| STAT-3 | *Mus musculus* | 1:2000 | BSA 5% | Cell Signaling Technology (9139) |
| $\alpha$-Tubulin | *Mus musculus* | 1:5000 | BSA 5% | Sigma-Aldrich (T6074) |

OSM, oncostatin M; BSA, bovine serum albumin.
