# Supplementary figures and images for: Impact of Liver Inflammation on Bile Acid Side Chain Shortening and Amidation
Source: Cells. 2022 Dec 9;11(24):3983. doi: 10.3390/cells11243983 (PMC9777420; doi:10.3390/cells11243983)

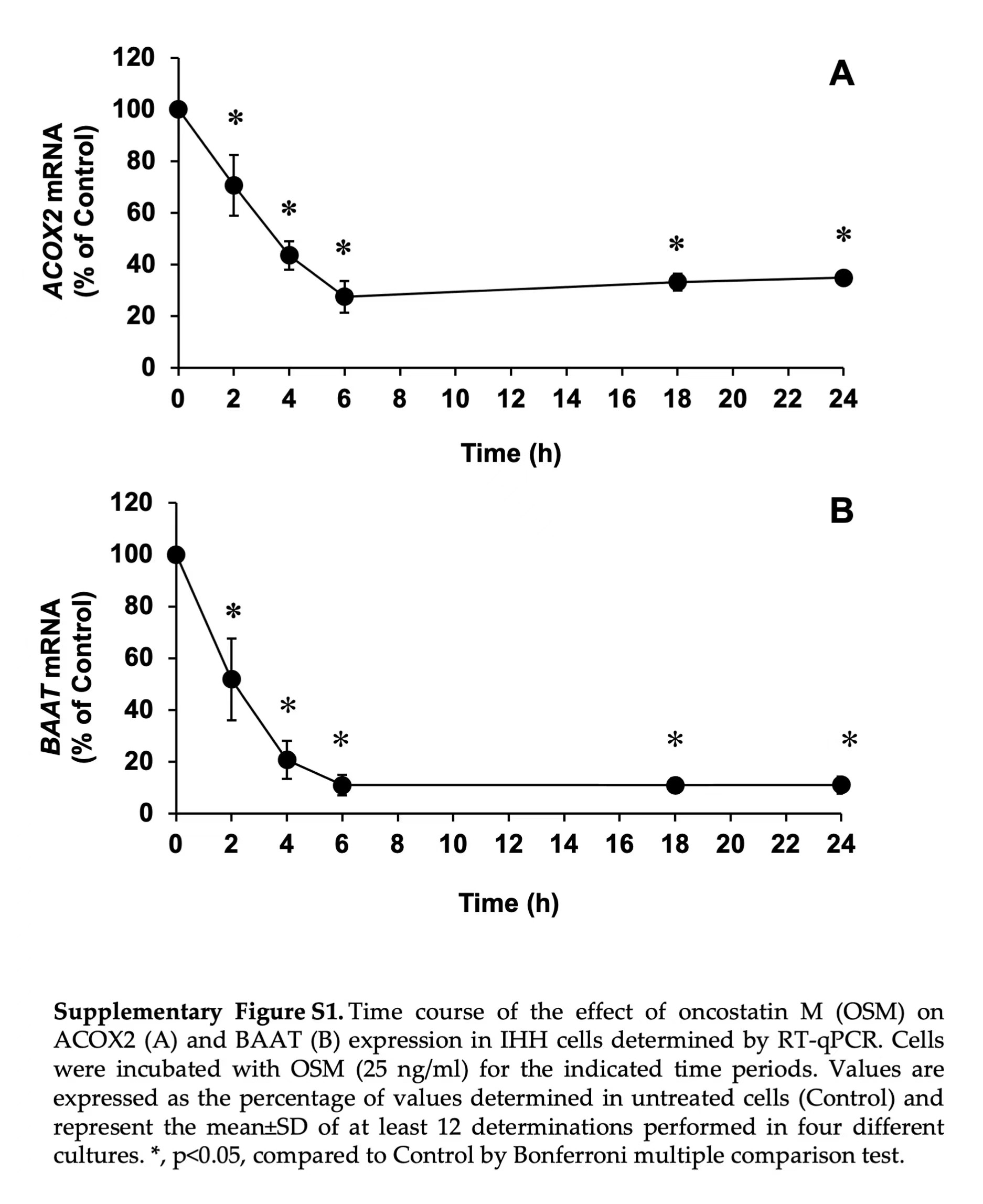

Supplement: Supplementary file 1 [file cells-11-03983-s001.zip › Figure S1.jpg]

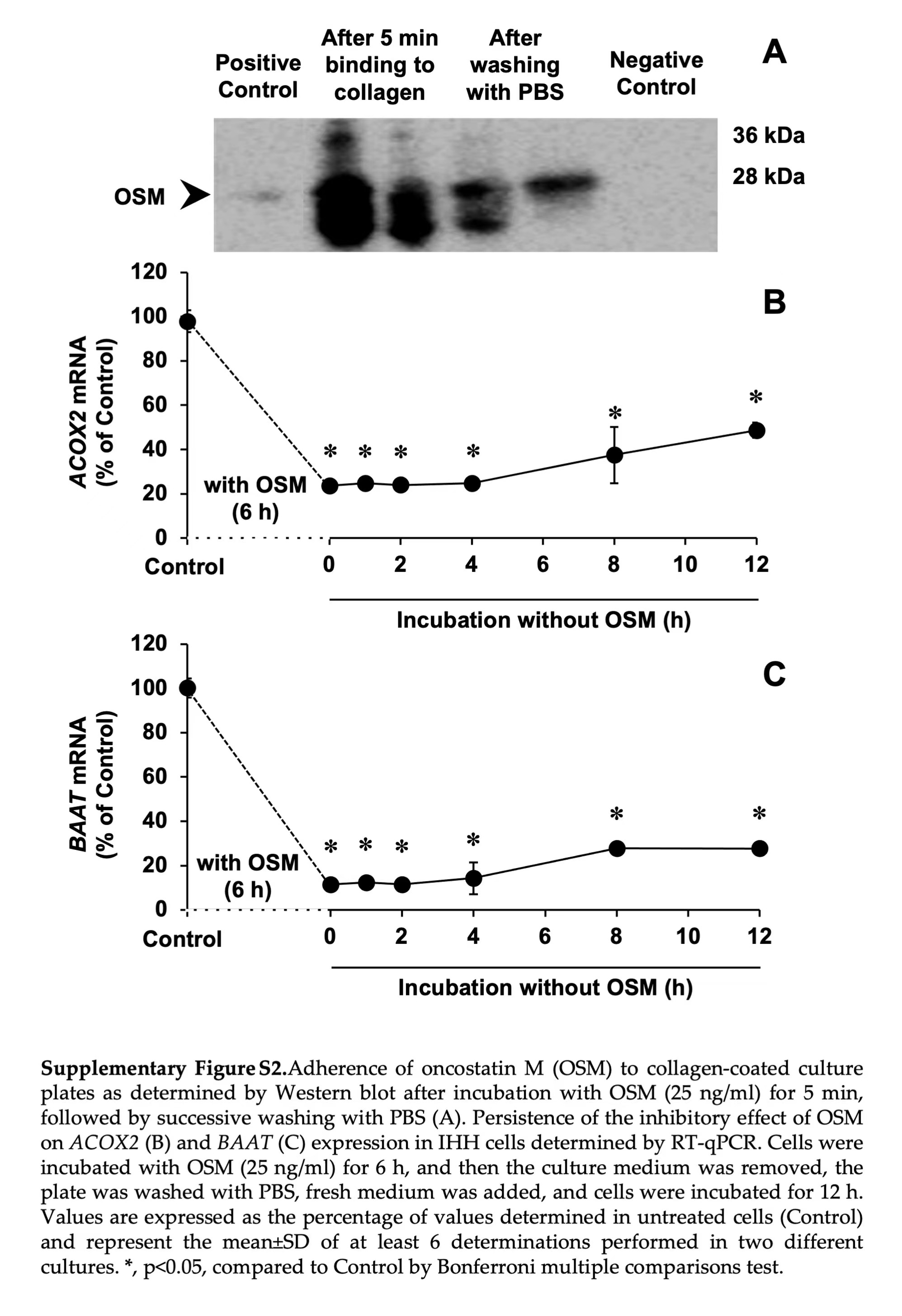

Supplement: Supplementary file 1 [file cells-11-03983-s001.zip › Figure S2.jpg]

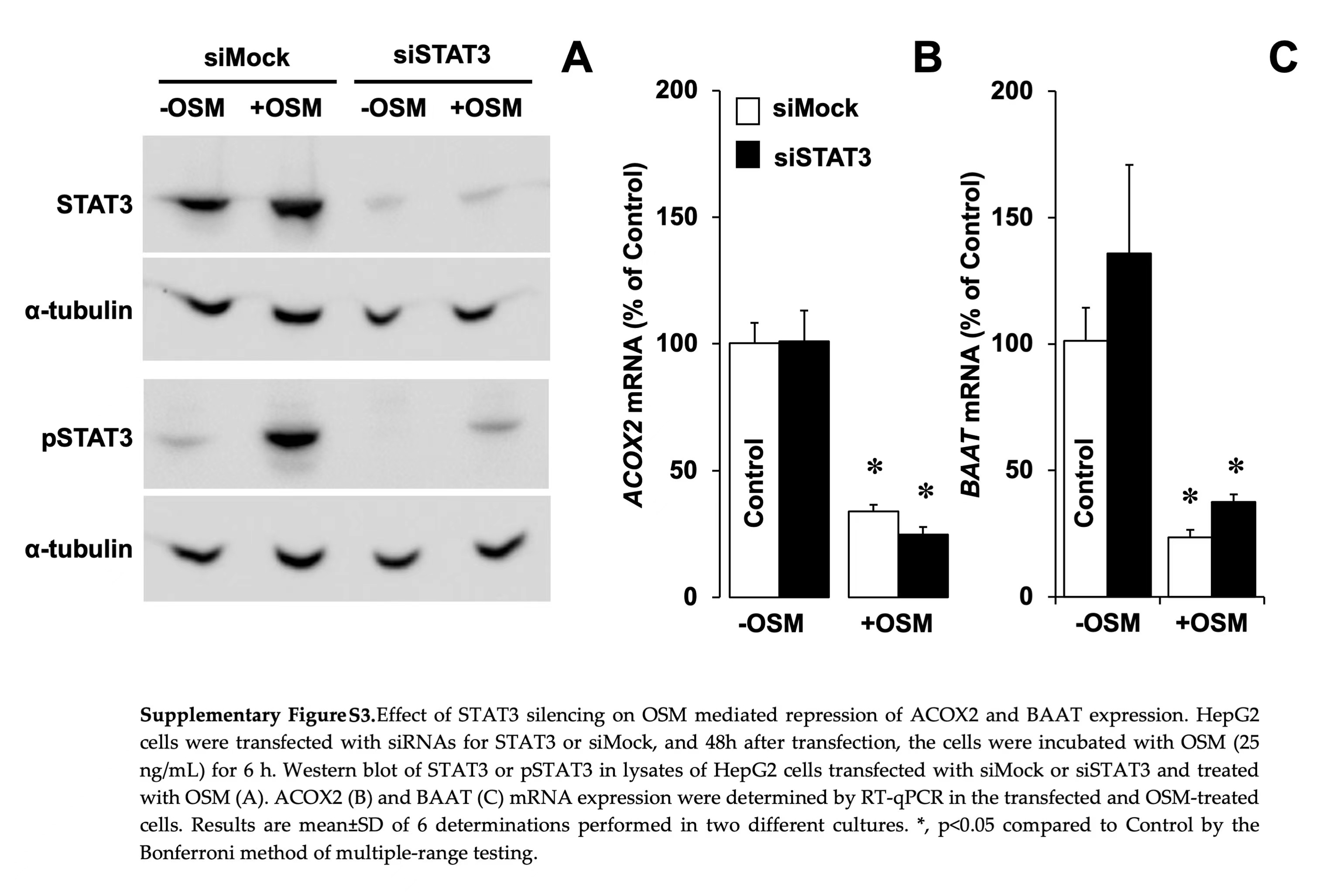

Supplement: Supplementary file 1 [file cells-11-03983-s001.zip › Figure S3.jpg]

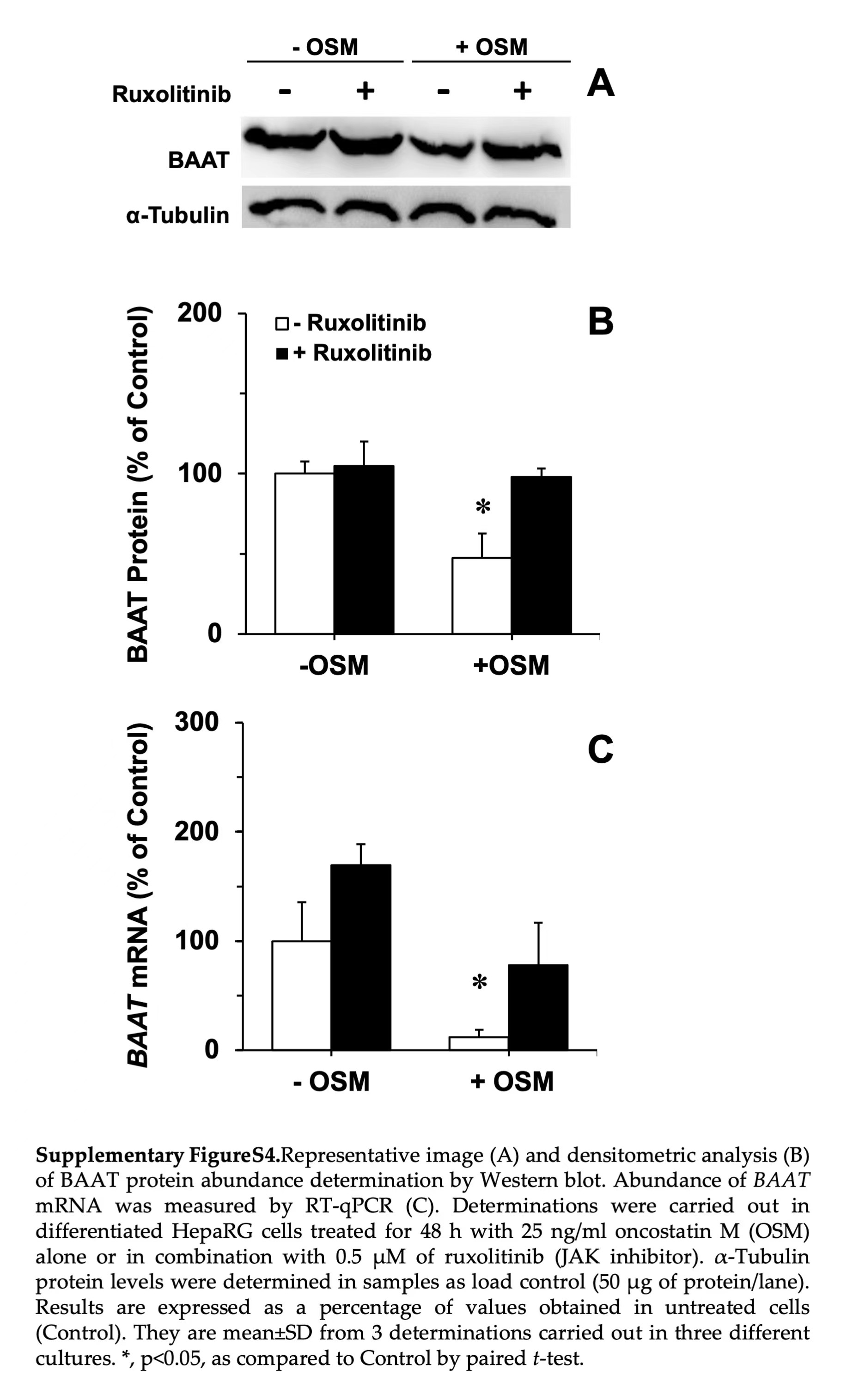

Supplement: Supplementary file 1 [file cells-11-03983-s001.zip › Figure S4.jpg]

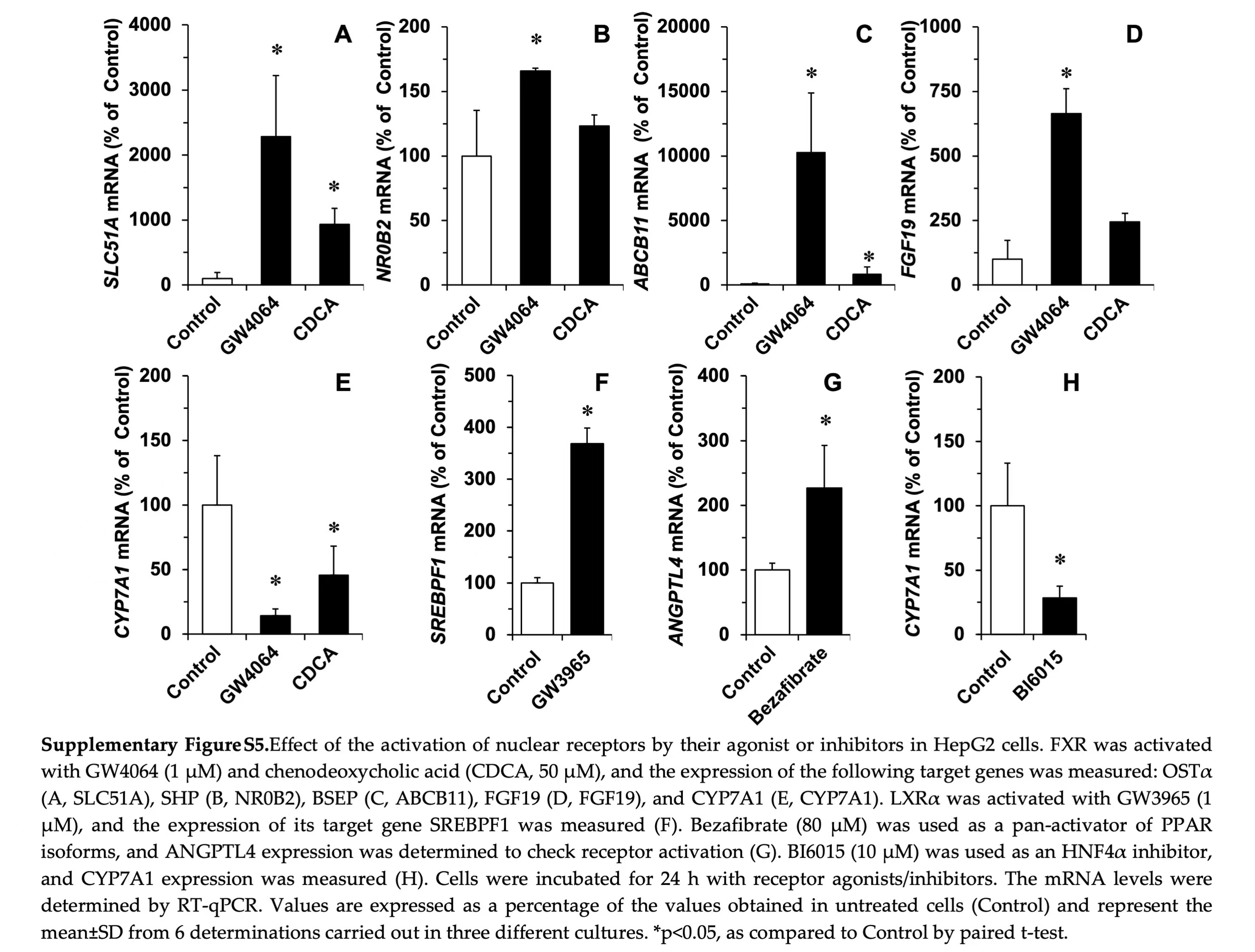

Supplement: Supplementary file 1 [file cells-11-03983-s001.zip › Figure S5.jpg]

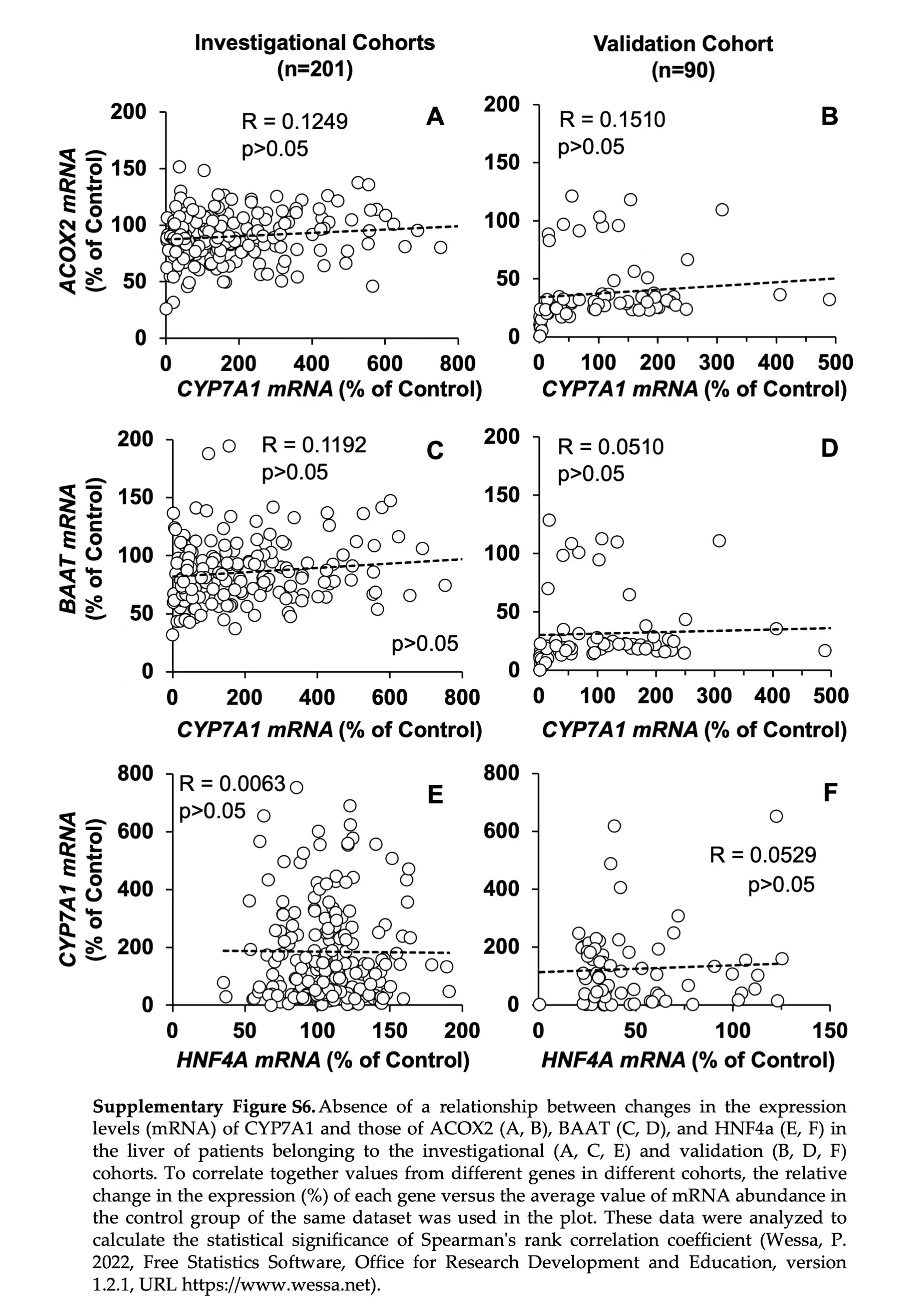

Supplement: Supplementary file 1 [file cells-11-03983-s001.zip › Figure S6.jpg]
